# Salience in Second Language Acquisition: Physical Form, Learner Attention, and Instructional Focus

**DOI:** 10.3389/fpsyg.2016.01284

**Published:** 2016-08-29

**Authors:** Myrna C. Cintrón-Valentín, Nick C. Ellis

**Affiliations:** ^1^Department of Psychology, University of Michigan, Ann ArborMI, USA; ^2^Department of Linguistics, University of Michigan, Ann ArborMI, USA; ^3^English Language Institute, University of Michigan, Ann ArborMI, USA

**Keywords:** second language acquisition, morphology, tense, learned attention, focus on form, grammar instruction, form-focused instruction, perceptual linguistic salience

## Abstract

We consider the role of physical form, prior experience, and form focused instruction (FFI) in adult language learning. (1) When presented with competing cues to interpretation, learners are more likely to attend to physically more salient cues in the input. (2) Learned attention is an associative learning phenomenon where prior-learned cues block those that are experienced later. (3) The low salience of morphosyntactic cues can be overcome by FFI, which leads learners to attend cues which might otherwise be ignored. Experiment 1 used eye-tracking to investigate how language background influences learners’ attention to morphological cues, as well as the attentional processes whereby different types of FFI overcome low cue salience, learned attention and blocking. Chinese native speakers (no L1 verb-tense morphology) viewed Latin utterances combining lexical and morphological cues to temporality under control conditions (CCs) and three types of explicit FFI: verb grammar instruction (VG), verb salience with textual enhancement (VS), and verb pretraining (VP), and their use of these cues was assessed in a subsequent comprehension test. CC participants were significantly more sensitive to the adverbs than verb morphology. Instructed participants showed greater sensitivity to the verbs. These results reveal attentional processes whereby learners’ prior linguistic experience can shape their attention toward cues in the input, and whereby FFI helps learners overcome the long-term blocking of verb-tense morphology. Experiment 2 examined the role of modality of input presentation – aural or visual – in L1 English learners’ attentional focus on morphological cues and the effectiveness of different FFI manipulations. CC participants showed greater sensitivity toward the adverb cue. FFI was effective in increasing attention to verb-tense morphology, however, the processing of morphological cues was considerably more difficult under aural presentation. From visual exposure, the FFI conditions were broadly equivalent at tuning attention to the morphology, although VP resulted in balanced attention to both cues. The effectiveness of morphological salience-raising varied across modality: VS was effective under visual exposure, but not under aural exposure. From aural exposure, only VG was effective. These results demonstrate how salience in physical form, learner attention, and instructional focus all variously affect the success of L2 acquisition.

## Introduction

### Psychological Aspects of Salience

Psychological research uses the term salience to refer to the property of a stimulus to stand out from the rest. Salient items or features are more likely to be perceived, to be attended to, and are more likely to enter into subsequent cognitive processing and learning. Salience can be independently determined by physics and the environment, and by our knowledge of the world. It is useful to think of three aspects of salience, one relating to psychophysics, the other two to what we have learned. (1) The physical world, our embodiment, and our sensory systems come together to cause certain sensations to be more intense (louder, brighter, heavier, etc.) than others. (2) As we experience the world, we learn from it, and our resultant knowledge values some associations higher than others. We know that some stimulus cues have affordances: they are associated with outcomes or possibilities that are important to us, while others are negligible (Gibson, 1977; James, 1890a, chap. 11). (3) We also have expectations about what is going to happen next in known contexts, we are surprised when our expectations are violated, and we pay more attention as a result. Each of the three phenomena is explained in more detail below.

#### Psychophysical Salience

Loud noises, bright lights, and moving stimuli capture our attention. Salience arises in sensory data from contrasts between items and their context. Stimuli with unique features compared to their neighbors (e.g., Os in a field of Ts, a red poppy in a field of yellow) “pop out” from the scene but in a shared feature context will not (Os among Qs; Treisman and Gelade, 1980). These are aspects of bottom-up processing (Shiffrin and Schneider, 1977).

#### Salient Associations

Attention can also be driven by top–down, memory dependent, expectation-driven processing. Emotional, cognitive, and motivational factors affect the salience of stimuli. These associations make a stimulus cue “dear.” A loved one stands out from the crowd, as does a stimulus with weighty associations ($500000.0 versus $0.000005, however, similar the amount of pixels, characters, or ink in their sensation). The units of perception are influenced by prior association: “The chief cerebral conditions of perception are the paths of association irradiating from the sense-impression, which may have been already formed” (James, 1890b, p. 82). Psychological salience is experience-dependent: *hotdog, sushi*, and 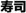 mean different things to people of different cultural and linguistic experience. This is why, *contra* sensation, the units of perception cannot be measured in physical terms. They are subjective. Hence George Miller’s definition of the units of short-term memory as “chunks”: “We are dealing here with a process of organizing or grouping the input into familiar units or chunks, and a great deal of learning has gone into the formation of these familiar units” (Miller, 1956, p. 91).

#### Context and Surprisal

The evolutionary role of cognition is to predict what is going to happen next, given that anticipation affords survival value. We find structure in time (Elman, 1990). The brain is a prediction machine (Clark, 2013). One consequence is that it is surprisal – when prediction goes wrong – that maximally drives learning from a single trial. Otherwise, the regularities of the usual course of our experiences sum little by little, trial after trial, to drive our expectations. Cognition is probabilistic, its expectations a conspiracy tuned from statistical learning over our experiences (Ellis, 2002).

### Salience and Learning

Rescorla and Wagner (1972) presented a formal model of conditioning which expresses the capacity of any cue [conditioned stimulus (CS), for example a bell in Pavlovian conditioning] to become associated with an outcome [unconditioned stimulus (US), for example food in Pavlovian conditioning] on any given experience of their pairing. This formula summarized over 80 years of research in associative learning, and it elegantly encapsulates the three factors of psychophysical salience, psychological salience, and surprisal. The role of US surprise and of CS and US salience in the process of conditioning can be summarized as follows:

dV=ab(L−V).

The associative strength of the US to the CS is referred to by the letter *V* and the change in this strength which occurs on each trial of conditioning is called *dV*. On the right hand side, *a* is the salience of the US, *b* is the salience of the CS, and *L* is the amount of processing given to a completely unpredicted US. Thus both the salience of the cue (a) and the psychological importance of the outcome (b) are essential factors in any associative learning. As for (L–V), the more a CS is associated with a US, the less additional association the US can induce. As Beckett (1954) put it: “habit is a great deadener.” Alternatively, with novel associations where V is close to zero, there is much surprisal, and consequently much learning: first impressions, first love, first time...

This is arguably the most influential formula in the history of learning theory. Physical salience, psychological salience, and surprisal interactively affect what we learn from our experiences of the world.

### Salience in Second Language Acquisition (SLA)

Naturalistic second language (L2) learners tend to focus more in their language processing upon open-class words (nouns, verbs, adjectives, and adverbs) than on grammatical cues. Their language attainment often stabilizes at a “Basic Variety” of interlanguage that predominantly comprises open-class words; closed-class items—in particular, grammatical morphemes and prepositions—are rare if present at all (Noyau et al., 1995).

Although naturalistic second language learners are surrounded by language *input*, the available target language, not all of it becomes *intake*, that subset of input that actually gets in and which the learner utilizes in some way (Corder, 1967). A classic case study is that of the naturalistic language learner, Wes, who was described as being very fluent, with high levels of strategic competence, but low levels of grammatical accuracy: “using 90% correct in obligatory contexts as the criterion for acquisition, none of the grammatical morphemes counted has changed from unacquired to acquired status over a 5 years period” (Schmidt, 1984, p. 5).

Although the Basic Variety is sufficient for everyday communicative purposes, grammatical morphemes and closed-class words tend not to be put to full use (e.g., Van Patten, 1996, 2006; Clahsen and Felser, 2006). Many untutored L2 learners initially make temporal references mostly by use of temporal adverbs, prepositional phrases, serialization, and calendric reference, with the grammatical expression of tense and aspect emerging only slowly thereafter, if at all (Meisel, 1987; Bardovi-Harlig, 1992, 2000; Noyau et al., 1995).

#### Psychophysical Salience

One factor determining the learning of cues is psychophysical salience: prepositional phrases, temporal adverbs, and lexical linguistic cues are salient and stressed in the speech stream. Verb inflections are not. In his landmark study of first language acquisition, Brown (1973, p. 343) breaks down the measurement of perceptual salience, or “clarity of acoustical marking,” into “such variables as amount of phonetic substance, stress level, usual serial position in a sentence, and so on” Brown (1973, p. 463).

Many grammatical form-function relationships in English, like grammatical particles and inflections such as the third person singular *-s*, are of low salience in the language stream. This is a result of the well-documented effect of frequency and automatization in the evolution of language. The basic principles of automatization that apply to all kinds of motor skills (like playing a sport or a musical instrument) are that through repetition, sequences of units that were previously independent come to be processed as a single unit or chunk (Ellis, 1996). The more frequently speakers use a form, the more they abbreviate it: this is a law-like relationship across languages. Zipf (1949) summarized this in the *principle of least effort* – speakers want to minimize articulatory effort so they tend to choose the most frequent words, and the more they use them, automatization of production causes their shortening. Frequently used words become shorter with use. Grammatical functors are the most frequent elements of a language, thus they lose their emphasis and tend to become abbreviated and phonologically fused with surrounding material (Bybee, 2000; Jurafsky et al., 2001; Zuraw, 2003).

Thus grammatical function words and bound inflections tend to be short and low in stress, with the result that these cues are difficult to perceive. In a corpus study by Cutler and Carter (1987), 86% of strong syllables occurred in open class words and only 14% in closed-class words; for weak syllables, 72% occurred in closed-class words and 28% in open-class words. When grammatical function words (*by, for, no, you*, etc.) are clipped out of connected speech and presented in isolation at levels where their open-class equivalents (*buy, four, know, ewe*, etc.) are perceived 90–100% correctly, adult native speakers can recognize them only 40–50% of the time (Herron and Bates, 1997). Clitics, accent-less words or particles that depend accentually on an adjacent accented word and form a prosodic unit together with it, are the extreme examples of this: the /s/ of ‘he’s’, /l/ of ‘I’ll’, and /v/ of ‘I’ve’ can never be pronounced in isolation.

In sum, grammatical functors are difficult to perceive from bottom-up auditory evidence alone. Fluent language processors can perceive these elements in continuous speech because their language knowledge provides top–down support. But this is exactly the knowledge that learners lack. Thus the low psychophysical salience of grammatical functors contributes to L2 learners’ difficulty in learning them (Goldschneider and DeKeyser, 2001; Ellis, 2006b).

#### Salience as Modulated by Modality

Spoken and written language are very different media, with spoken language being fleeting while writing provides more permanent visual substance on the page, allowing the reader to attend linguistic form at their discretion. Attention to language form may therefore pose different challenges in written and spoken modalities. VanPatten (1990) showed that L2 learners of Spanish have difficulty simultaneously attending to meaning and form of aural input. He had them process spoken Spanish passages for meaning while simultaneously monitoring the input for either lexical content words like *inflacíon* or for grammatico-morphological forms like the definite article *la* or the verb morpheme *–n*. Monitoring grammatico-morphological forms negatively affected comprehension, whereas attention to lexical items did not. Wong (2001) replicated this study while also adding conditions in the written modality. She showed that comprehension was worse from aural language than from written language. Furthermore, while the results from the aural conditions replicated the patterns found by VanPatten, the number of idea units recalled by readers who had to pay attention to the definite article in the written input was not significantly less than those who read the passage for content only or for those who had to attend to the lexical item *inflacíon.* Thus modality can differentially affect the salience of forms and their input processing: written language can make grammatical forms more salient and more easily processed.

#### Learned Attention

In addition to psychophysical factors, there are attentional factors which affect the salience of grammatical functors. The first relates to their redundancy. Grammatical morphemes often appear in redundant contexts where their interpretation is not essential for correct interpretation of the sentence (Terrell, 1991; Van Patten, 1996; Schmidt, 2001). Tense markers often appear in contexts where other cues have already established the temporal reference (e.g., “*yesterday he walked*”), plural markers are accompanied by quantifiers or numerals (“*27 cats*”), etc. Hence their neglect does not result in communicative breakdown, they carry little psychological importance of the outcome (term *b* in the Rescorla-Wagner equation), and the Basic Variety satisfices (Simon, 1957) for everyday communicative purposes.

Still again, there are attentional biases that result from L2 learners’ history of learning – from their knowledge of a prior language. Ellis (2006a,b) attributes L2 difficulties in acquiring inflectional morphology to an effect of *learned attention* known as *blocking* (Kamin, 1969; Mackintosh, 1975; Kruschke and Blair, 2000; Kruschke, 2006). Blocking is an associative learning phenomenon, occurring in animals and humans alike, that shifts learners’ attention to input as a result of prior experience (Rescorla and Wagner, 1972; Shanks, 1995; Wills, 2005). Knowing that a particular stimulus is associated with a particular outcome makes it harder to learn that another cue, subsequently paired with that same outcome, is also a good predictor of it. The prior association “blocks” further associations.

All languages have lexical and phrasal means of expressing temporality. So anyone with knowledge of any first language is aware that that there are reliable and frequently used lexical cues to temporal reference (words like German *gestern*, French *hier*, Spanish *ayer*, English *yesterday*). Such are cues to look out for in an L2 because of their frequency, their reliability of interpretation, and their salience. Learned attention theory holds that, once known, such cues block the acquisition of less salient and less reliable verb tense morphology from analysis of redundant utterances such as *Yesterday I walked*. The Input Processing (IP) theory of SLA (Van Patten, 1996) includes a *Lexical Preference Principle*: “Learners will process lexical items for meaning before grammatical forms when both encode the same semantic information” (Van Patten, 2006, p. 118), and a *Preference for Non-redundancy Principle*: “Learners are more likely to process non-redundant meaningful grammatical markers before they process redundant meaningful markers” (Van Patten, 2006, p. 119).

Summing up, grammatical functors abound in the input, but, as a result of their low salience, their redundancy, the low contingency of their form-function mappings, and adult acquirers’ learned attentional biases and L1-tuned automatized processing of language, they are simply not implicitly learned by many naturalistic learners whose attentional focus is on communication.

### Prior Experiments on Learned Attention and Blocking in SLA

Ellis and Sagarra (2010, 2011) and Ellis et al. (2014) report a series of experimental investigations of learned attention in SLA involving the learning of a small number of Latin expressions and their English translations. We sketch them in some detail here because they introduce key concepts and because we build on their design in the present study.

In Ellis and Sagarra (2011) there were three groups: Adverb Pretraining, Verb Pretraining, and Control. In Phase 1, Adverb Pretraining participants learned two adverbs and their temporal reference – *hodie* today and *heri* yesterday; Verb Pretraining participants learned verbs (shown in either first, second, or third person) and their temporal reference – e.g., *cogito* present or *cogitavisti* past; the Control group had no such pretraining. During Phase 2, Sentence Exposure, all participants were shown sentences which appropriately combined an adverb and a verb (e.g., *heri cogitavi, hodie cogitas, cras cogitabis*) and learned whether these sentences referred to the past, the present, or the future. In Phase 3, the Reception test, all combinations of adverb and verb tense marking were presented individually and participants were asked to judge whether each sentence referred to the past, present, or future. The logic of the design was that in Phase 2 every utterance contained two temporal references – an adverb and a verb inflection. If participants paid equal attention to these two cues, then in Phase 3 their judgments should be equally affected by them. If, however, they paid more attention to adverb (/verb) cues, then their judgments would be swayed toward them in Phase 3.

The Control Group illustrate the normal state of affairs when learners are exposed to utterance with both cues and learn from their combination. Multiple regression analysis, where the dependent variable was the mean temporal interpretation for each of the Phase 3 strings and the independent variables were the information conveyed by the adverbial and verbal inflection cues showed in standardized ß coefficients, Control Group Time = 0.93 Adverb + 0.17 Verb. The adverb cues far outweighed the verbal inflections in terms of learnability. We believe this is a result of two factors (i) the greater salience of the adverbial cues, and (ii) learned attention to adverbial cues which blocks the acquisition of verbal morphology.

The two other groups reacted to the cues in quite different ways – the Adverb pretraining group followed the adverb cue, the Verb pretraining group tended to follow the verb cue: Adverb Group Time = 0.99 Adverb – 0.01 Verb; Verb Group Time = 0.76 Adverb + 0.60 Verb. Pretraining on the verb in non-redundant contexts did allow acquisition of this cue when its processing was task-essential, but still, the adverb predominated.

Ellis and Sagarra (2010, Experiment 2) and Ellis and Sagarra (2011, Experiments 2 and 3) also illustrated long-term language transfer effects whereby the nature of learners’ first language (+/- verb tense morphology) biased the acquisition of morphological versus lexical cues to temporal reference in the same subset of Latin. First language speakers of Chinese (no tense morphology) were less able than first language speakers of Spanish or Russian (rich morphology) to acquire inflectional cues from the same language experience where adverbial and verbal cues were equally available, with learned attention to tense morphology being in standardized ß coefficients: Chinese (-0.02) < English (0.17) < Russian (0.22) < Spanish (0.41) (Ellis and Sagarra, 2011, p. 612). These findings suggest that there is a long-term attention to language, a processing bias affecting subsequent cue learning that comes from a lifetime of prior L1 usage.

### Enhancing Attention to Non-salient Forms: The Role of Form-Focused Instruction

Several theories of SLA (e.g., Schmidt, 2001; Ellis, 2005) emphasize the centrality of attention. Schmidt’s (2001) *Noticing Hypothesis* holds that conscious attention to linguistic forms in the input is an important precondition to learning: “people learn about the things they attend to and do not learn much about the things they do not attend to” (Schmidt, 2001, p. 30).

Form focused instruction (FFI) attempts to encourage noticing, drawing learners’ attention to linguistic forms that might otherwise be ignored (Spada, 1997; Spada and Tomita, 2010; Ellis, 2012). Variants of FFI vary in the degree and manner in which they recruit learner consciousness and in the role of the learner’s metalinguistic awareness of the target forms (Ellis, 1994; Rebuschat, 2015). Explicit instruction traditionally centers upon “some sort of rule being thought about during the learning process” (DeKeyser, 1995). This type of instruction can be deductive, when learners are presented with grammar rule explanation, or inductive, when they are asked to attend to a particular set of forms with the purpose of inferring the rules on their own. This may include explicit metalinguistic feedback, which provides “comments, information, or questions, related to the well-formedness of the learner’s utterance” (Lyster and Ranta, 1997, p. 47). Conversely, through more implicit instruction, learners are expected to infer regularities of form-meaning patterns without awareness. Having laid out the bare contrast like this, we emphasize that there is no simple binary divide between explicit and implicit instruction, that implicit and explicit knowledge interact, and that this is still an area of considerable research inquiry (e.g., Ellis, 1994, 2005; Rebuschat, 2015).

Long (1991) and Doughty and Long (2003) describe how a focus on meaning can be improved upon by periodic attention to language as object: during otherwise meaning-focused lessons, learners’ attention is briefly shifted to linguistic code features, in context, to induce noticing. This is known as focus-on-form. Doughty and Williams (1998) give the following examples of focus-on-form techniques, ranging from less to more explicit: input flood, where texts are saturated with L2 models; input elaboration; input enhancement, where learner attention is drawn to the target through visual highlighting or auditory stress; corrective feedback on error, such as recasting; and input processing, where learners are given practice in using L2 rather than L1 cues.

Norris and Ortega’s (2000) meta-analysis comparing the outcomes from studies that employed differing levels of explicitness of L2 input demonstrated that FFI instruction results in substantial target-oriented L2 gains, that explicit types of instruction are more effective than implicit types, and that the effectiveness of L2 instruction is durable. More recent meta-analyses of effects of type of instruction by Spada and Tomita (2010) and Goo et al. (2015) likewise report large advantages of explicit instruction in L2 acquisition. However, the studies gathered in these meta-analyses used a wide variety of types of instruction, learner, targeted feature, and method of assessment. There is need to compare FFI methods upon the processing of the same target feature in similar populations of learners.

This is one of the aims of our current study, which employs a series of explicit and implicit FFI techniques to contrast and illuminate the processes by which these different methods help learners refocus their attention to non-salient forms in the input. In the following sections we will discuss and operationalize the different types of FFI included in our design: (1) Verb grammar (VG), (2) Textual enhancement (TE), and (3) Verb pretraining (VP) in isolation in task-essential rather than redundant contexts.

#### Verb Grammar (VG)

One method that has been widely investigated both in SLA research and practice is that of explicit grammar instruction (EGI) which Terrell (1991, p. 53) defines as “the use of instructional strategies to draw the students’ attention to, or focus on, form and/or structure,” with instruction targeted at increasing the salience of inflections and other commonly ignored features by, first, pointing them out and explaining their structure and, second, providing meaningful input that contains many instances of the same grammatical meaning-form relationship. Ellis (2006) reviews studies of EGI demonstrating that learning through explicit means alone, that is, without the provision of tasks requiring the learner to practice the target features before being tested on their knowledge of these forms, seems to be ineffective (e.g., Ellis, 1993; VanPatten and Oikennon, 1996). We therefore operationalized VG as short metalinguistic description of simple regular tense morphology in Latin which was followed by a sentence exposure phase where leaners were presented with phrases combining adverbs and verb cues to temporality and were asked to determine the appropriate tense before proceeding to comprehension.

#### Textual Enhancement

Another common FFI technique is the use of Textual Enhancement such as color-coding, boldfacing and underlining, to increase learners’ awareness of non-salient forms in the input (Sharwood-Smith, 1993; Doughty and Williams, 1998). Han et al. (2008) and Lee and Huang (2008) review studies of TE and conclude that there are conflicting findings regarding its effectiveness. They suggest that these discrepancies may be explained by differences between studies in such factors as learners’ target and native languages, the type, complexity and communicative value of target forms, learner proficiency, treatment intensity, and the measures used to assess noticing and processing of these forms.

In the present study, we used boldfacing and color to make verb-tense inflections more salient. This condition is therefore called verbal salience (VS). Contrary to the VG condition, we did not explicitly direct learners to attend to the enhanced verb-inflections. Nevertheless, given that we did provide VS participants with explicit feedback on their correctness during the exposure phase, we consider VS an explicit FFI technique designed to promote induction of the target form.

#### Verb Pretraining

The effect of blocking is particularly potent whenever the cue to be processed is met in a redundant context where other cues have the same interpretation and have been learned previously or are more salient. One way to counteract this type of blocking is to ensure that early in L2 experience, the cue is experienced on its own in situations in which it must be processed for successful interpretation (VanPatten and Oikennon, 1996). Ellis and Sagarra’s (2010, 2011) VP conditions tested the effects of this and demonstrated that once the cue has been consolidated into the processing system, it continues to contribute to processing in subsequent situations of potential cue competition. For continuity, replication, and comparison, we include VP here to compare its efficiency and operation with VG and VS conditions. VP does not explicitly provide learners with a metalinguistic description of verb-tense morphology, but rather gives them opportunity to infer how verb tense morphology works by processing Latin verb forms for temporality and providing feedback on their correctness.

### Eye-Tracking as a Measure of Attention

Second Language Acquisition research is increasingly recognizing eye tracking as a research tool (Winke et al., 2013) because it “allows for the study of moment-by-moment processing decisions during natural, uninterrupted comprehension, and critically, without the need to rely on participants’ strategic or metalinguistic responses” (Roberts and Siyanova-Chanturia, 2013, p. 214). Ellis et al. (2014) used eye movement recordings to measure participants’ overt attention to adverb and verb cues and found that pretraining on different cue dimensions (adverb pretraining versus verb pretraining) led to differences in learners’ overt attention to these cues during processing, and that these in turn led to differences in their covert attention to these cues during the comprehension and production tasks.

### Aims

The current studies extend previous research on salience and learned attention in SLA by (i) exploring and comparing the degree to which VG, VS, and VP methods of FFI might serve to counteract learned attention effects whereby learners’ prior experience with adverbial cues in their L1 block their processing of verb inflections in the L2, and (ii) comparing their effects in aural and visual modalities of language.

In Experiment 1 we use eye-tracking to measure Chinese L1 speakers’ visual attention to form in these various FFI conditions of visual language exposure. The control condition (CC) and VP conditions allow us to replicate Ellis and Sagarra (2011), as well as to extend the findings in Ellis et al. (2014) using a more complex verbal system. The inclusion of VG and VS, additionally allow us to further compare the effects of these manipulations to VP.

Experiment 1 focuses upon several research questions:

*Research Question 1* (RQ1): do the effects of physical salience and learned attentional biases toward adverbial cues, under normal conditions of exposure (CC), prejudice the acquisition of verbal tense morphology, as indexed in participants’ relative reliance on these cues in subsequent language comprehension?
*Research Question 2* (RQ2): does early experience of morphological cues to temporal reference, through each of the FFI treatments VG, VS, VP, counteract the effects of physical salience and learned attentional biases, as indexed by participants’ relative reliance on these cues in subsequent language comprehension?
*Research Question 3 (RQ3):* does early experience of morphological cues to temporal reference lead to biases in subsequent overt perceptual attention (as indexed by number of fixations) during Sentence Exposure, where there are both adverbial and morphological cues to the same interpretation?
*Research Question 4 (RQ4):* does any bias in overt attention to input cues in turn lead to subsequent attentional biases to the adverbial or morphological cues in subsequent language comprehension?

In Experiment 2 we compare the processing of auditory and visual input to assess effects of modality on salience, and again we contrast the effectiveness of VG, VS and VP methods of FFI in counteracting learned attention effects. The research questions of Experiment 2 are:

*Research Question 5 (RQ5):* as in Experiment 1, does early experience of morphological cues to temporal reference, through each of the FFI treatments VG, VS, and VP, counteract the effects of physical salience and learned attentional biases, as indexed by participants’ relative reliance on these cues in subsequent language comprehension.
*Research Question 6 (RQ6):* are each of the FFI treatments VG, VS, and VP equally effective in reattuning learners’ attention to the non-salient morphological cues through visual and auditory modalities of exposure?

## Experiment 1

### Introduction

Cintrón-Valentín and Ellis (2015) used eye-tracking to investigate the attentional processes whereby different types of FFI instruction overcome learned attention and blocking effects in learners’ online processing of L2 input. English native speakers viewed Latin utterances combining lexical and morphological cues to temporality under control conditions (CC) and three types of explicit FFI: verb grammar instruction (VG), verb salience with textual enhancement (VS), and verb pretraining (VP). All groups participated in three phases: exposure, comprehension test, and production test. VG participants viewed a short lesson on Latin tense morphology prior to exposure. VS participants saw the verb inflections highlighted in bold and red during exposure. VP participants had an additional introductory phase where they were presented with solitary verb forms and trained on their English translations. Instructed participants showed greater sensitivity to morphological cues in comprehension and production testing. Eye-tracking measures revealed how FFI affects learners’ attention during online processing and thus modulates long-term blocking of verb morphology.

This experiment aims to replicate these findings in another population of learners, L1 Chinese speakers, whose L1 does not exhibit verb-tense morphology. In Chinese languages, “gender, plurality and tense are either indicated by lexical choice or not indicated at all” (Li and Thompson, 1987, p. 825). As a result, L1 speakers of Chinese languages are particularly prone to long-term attentional blocking of verb tense morphology (Ellis and Sagarra, 2011).

### Participants

Chinese native speakers who had not learned Latin or Italian previously were recruited from a major university in the USA (*n* = 58) or its local community (*n* = 9). They were volunteers and either participated as part of an undergraduate Psychology course requirement (*n* = 3) or they were compensated with 10 dollars for their time (*n* = 64). All were bilingual with high-level English language proficiency sufficient to admit them to study in English. However, all had learned English as a L2 after the age of 5 years. They were randomly assigned to one of four conditions: CC, *n* = 19 (12 females and 7 males), age range 19–35 years (*M* = 24.58); VG, *n* = 18 (13 females and 5 males), age range 20–26 years (*M* = 22.50); VS, *n* = 14 (11 females and 4 males), age range 19–30 years (*M* = 23.13) and; VP, *n* = 15 (10 females and 4 males), age range 20–34 years (*M* = 24.80). Of these participants, seven (CC = 4; VG = 2; VS = 1) were excluded from the eye-tracking analyses due to poor data quality. All participants received oral instructions in their native language prior to the start of the experiment, with the exception of three participants in the Chinese CC group and four participants in the Chinese VG group. Although it was originally intended that all participants would receive these additional instructions in their native language, to ensure that they were indeed bilingual, the research assistants were not all fluent in Chinese.

### Procedure

The experiment was programmed in E-Prime (Schneider et al., 2002). It took less than 1 h to complete. There were three phases: Pretraining, Sentence Exposure, and Comprehension testing. The procedure of these phases is shown in **Table 1**.

**Table 1:** The design of Phases 1–3 of Experiment 1.

Pretraining (Phase 1) (+ feedback)	Sentence Exposure (Phase 2) (+ feedback) 36 (18 × 2) randomized trials	Comprehension Test (Phase 3) (- feedback) 66 randomized trials	
		Stimulus	Semidiem
**Control group**	**Present**		
No pretraining **Verb Pretraining group** (36 randomized trials) *cogito* “I think” *cogitas* “you think” *cogitat* “X thinks” *cogitavi* “I thought” *cogitavisti* “you thought” *cogitavit* “X thought” **Verb Salience group** No pretraining **Verb Grammar group** Brief Grammar Lesson See **Figure 1**	*hodie cogito* *hodie cogitas* *hodie cogitat* *cogito hodie* *cogitas hodie* *cogitat hodie* **Past** *heri cogitavi* *heri cogitavisti* *heri cogitavit* *cogitavi heri* *cogitavisti heri* *cogitavit heri* **Future** *cras cogitabo* *cras cogitabis* *cras cogitabit* *cogitabo cras* *cogitabis cras* *cogitabit cras*	*hodie* *heri* *cras* *cogito/as/at* *cogitavi/visti/vit* *cogitabo/bis/bit* *hodie cogito/as/at* *hodie cogitavi/visti/vit* *hodie cogitabo/bis/bit* *heri cogito/as/at* *heri cogitavi/visti/vit* *heri cogitabo/bis/bit* *cras cogito/as/at* *cras cogitavi/visti/vit* *cras cogitabo/bis/bit* *cogito/as/at hodie* *cogitavi/visti/vit hodie* *cogitabo/bis/bit hodie* *cogito/as/at heri* *cogitavi/visti/vit heri* *cogitabo/bis/bit heri* *cogito/as/at cras* *cogitavi/visti/vit cras* *cogitabo/bis/bit cras*	3 1 5 3 1 5 3 2 4 2 1 3 4 3 5 3 2 4 2 1 3 4 3 5

*The rating scale for the Comprehension Test ranged from 1 (*past*) to 5 (*future*). The correct answer for each trial is shown in the semidiem column.*

#### Pretraining

Verb pretraining participants engaged in a phase that involved training on verb inflections. On each trial they saw one of the past (*cogitavi, cogitavisti, cogitavit*) or present (*cogito, cogitas, cogitat*) inflected verbs and learned that each corresponded to either X think(s) or X thought by clicking the appropriate alternative with the mouse. A correct choice returned the feedback “Correct” or “Incorrect – the meaning of [Latin word] is [English word].” The 36 trials thus involved each of the three persons singular of present and past tense being presented six times in random order. Keeping the same number of trials of pretraining for all participants allows evaluation of what is gained from that amount of experience. This permits comparison across contents and conditions of pretraining, for example auditory versus visual modality as in Experiment 2 which follows here, and those of Ellis and Sagarra (2010, 2011) which vary with regard to the different levels of grammatical number and person. We report performance levels at the end of training in 2.4.1.

**FIGURE 1:**
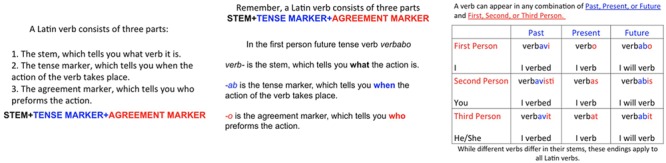
**Grammar Lesson Slides for Grammar Instruction Condition**.

Pretraining for the VG participants involved a brief lesson on Latin verb-tense morphology using the three slides shown in **Figure 1**. Although they could view each of the slides for an undetermined amount of time, they were not allowed to take notes and could not regress to previous slides.

#### Sentence Exposure

**FIGURE 2:**
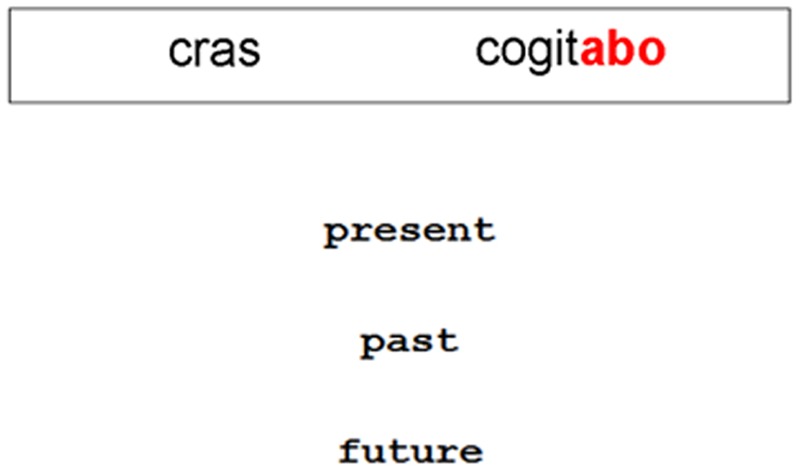
**An example trial from the Verb Salience Condition, where the verb inflections were highlighted in bold and red during Sentence Exposure**.

During Sentence Exposure, participants were exposed to 18 sentences (see **Table 1**) that appropriately combined the adverb with a verb (half in adverb-verb word order and half in verb-adverb order) and had to choose whether these sentences referred to the present, the past, or the future. Both word orders were used to counterbalance which cue was experienced first across sentences. Each of the 18 sentences was presented twice during this phase of the experiment. Feedback was given for both correct and incorrect choices. For correct answers, the word “correct” would appear on the screen, whereas for incorrect answers, participants would see the word “wrong” accompanied by the correct answer (e.g., “Wrong – [*heri cogitavisti*] is [past]”). The Sentence Exposure procedure was identical for the CC, VG, and VP groups. For VS participants only, the stimuli were textually enhanced so that the verbal inflections were highlighted in bold and red to increase the salience of these items (see **Figure 2**). Participants were not made aware of this beforehand and were given the same instructions for this task as were the other groups.

#### Comprehension Test

In this phase, participants were presented with all single-word items (verbs and adverbs) and all possible combinations of adverbs and verb tenses for a total of 12 single-word items and 54 two-word items (comprised of 27 unique combinations), respectively (a grand total of 66 trials; see **Table 1**). The two-word items were presented in two different word orders, counterbalancing the cue participants would experience first. The presentation of all possible combinations meant that participants experienced sentences that were familiar to them from the previous task and also combinations in which the verb and adverb were incongruent in their time reference. Before the start of the task participants were told that there would be both congruent and incongruent sentences. They were asked to judge their temporal reference on a five-point scale by using their mouse to select the appropriate answer. The possible scale points were labeled (1) “past,” (2) “both past and present,” (3) “present,” (4) “both present and future,” and (5) “future.” Participants were told they could also choose 3 if they encountered an incongruent sentence with both past and future cues. For example, the participant could be presented with an incongruent sentence such as *heri cogitabo* “Yesterday I will think,” for which the correct answer was 3 and understood as the average of the items’ tenses (past [1] + future [5]/2 = 3). The correct answer for each trial, which Ellis and Sagarra (2011) referred to as the semidiem, is shown in **Table 1**. This task separately assessed the degree to which participants attended the adverb and verbal cues by determining the relative weight that learners put on adverbial and inflectional cues to time reference. For this reason, feedback was not provided.

The logic behind the experiment follows that of previous studies of learned attention and blocking (Ellis and Sagarra, 2010, 2011; Ellis et al., 2014). During Sentence Exposure, regardless of condition, all participants experience both the adverb and verbal cue together. If they pay equal attention to both cues during this phase then their judgment during the Comprehension test should be equally affected by both cues. However, if they are biased toward one cue or the other, it is expected that their judgment in the Comprehension test will be swayed toward the corresponding cue. Because the CC participants only saw the two cues together, their performance was expected to mirror how learners typically weigh these cues, which in the native speakers of English studied in Ellis and Sagarra (2010, 2011) was characterized by the overshadowing of morphological cues by the more salient and reliable adverbial cues.

#### Eye-Tracking

Eye-movement recordings were gathered using an ISCAN-ETL 400 eye-imaging system with a sampling rate of 60 Hz. The eye-tracking cameras were mounted on headgear. Before the start of Pretraining (or Sentence Exposure for the CC and VS participants), the participants’ gaze was calibrated using a six-point calibration sequence. This sequence was again repeated for all participants before starting Comprehension testing. Stimuli were presented in E-Prime and were positioned within a screen area of 640 × 480 pixels. In the Sentence Exposure phase, the left stimulus (STIML) was centered at coordinates (x, y) 94, 99, and the right stimulus (STIMR) was positioned at coordinates 454, 99. For Comprehension testing, STIML and STIMR were positioned at 109, 108 and 505, 108, respectively. Participants’ fixations were analyzed using ILAB (Version 3.6.4), an open-source program developed for the analysis of eye-movement recordings (Gitelman, 2002) through the MATLAB software platform (Version 7.12.0.635) (MathWorks Inc, 2011). For each condition, fixations were analyzed from 600 ms after the start of Sentence Exposure and Comprehension testing trials (coinciding with the end of the presentation of a fixation cross at the center of the screen) until the end of each trial (coinciding with participant response). Region of interest (ROI) analyses were calculated using two positions (left and right) at the upper-most part of the screen. Both ROIs had a height of 200 pixels and a width of 250 pixels; the ROI for STIML was centralized at 175, 103 pixels and the ROI for STIMR, at 465, 103 pixels. These relatively large ROIs reflect our simple setup, which involved merely a chin rest and forehead bar to stabilize the participant’s head position. In some cases, for individual subjects it was necessary to edit coordinates for both ROIs to adjust for drift. Fixation analyses were run using the default ILAB fixation velocity/distance calculation parameters, with fixations determined according to degree of movement (horizontal 1.02°; vertical 1.09°) and a minimum duration of 100 ms. Eye-movement analysis was done blind to stimulus content: the random order of stimulus presentation for each participant entailed that right and left fixation durations were assigned as verb and adverb fixation durations only in subsequent statistical analysis on the basis of trial number.

## Results

### Behavioral Data

#### Verb Pretraining Data

Mean performance in the first quarter of Verb Pretraining was 79%. By the fourth quarter, mean performance was 93% (nine participants attained 100%, five 89%, and one 56%) demonstrating that the amount of training in Phase 1 was at an appropriate level.

#### Sentence Exposure Data

Mean performance in the first quarter of Sentence Exposure was 60% for the CC group, 62% for the VG group, 49% for the VS group, and 74% for the VP group: the prior experience of VP participants gave them an advantage in the first quarter compared to the other groups. However, performance evened out for all groups by the end of the phase. Mean performance in the final quarter was 82% for the CC group, 84% for the VG group, 73% for the VS group, and 89% for the VP group. A one-way ANOVA on these final quarter scores did not reveal a significant group effect, *F*(3,63) = 1.55, *p* = 0.21.

#### Comprehension Data

**FIGURE 3:**
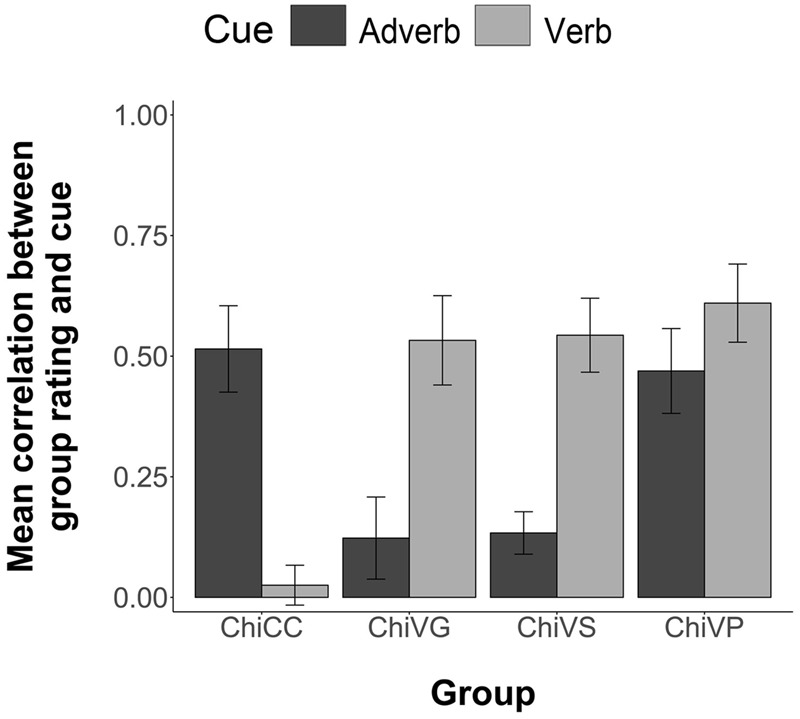
**Group mean correlations between individual participants’ Comprehension sentence ratings and the information given by the corresponding adverb and verb cues.** Error bars are 2 standard errors long. Chi = Chinese.

For each participant, we computed the Pearson correlation between the temporal ratings they provided for each of the 54 two item stimuli in the comprehension phase and the information given in each sentence by the corresponding adverb and verb cues. This correlation thus shows the degree to which each participant is biased by the verb and adverb cues. **Figure 3** illustrates the group mean correlations. Following Corey et al. (1998), when averaging or performing inferential statistics on the correlation coefficients, we first transformed the *r* values to *z* values, then performed the statistics, and then reverse transformed to report the values. Participants in the four groups differed in their cue use. Chinese CC participants were more influenced by the adverb, *M* = 0.51, 95% CI = [0.34, 0.69] than the verb, *M* = 0.03, 95% CI = [-0.06, 0.11]. Chinese Verb grammar participants were more influenced by the verb, *M* = 0.53, 95% CI = [0.35, 0.71], than by the adverb, *M* = 0.13, 95% CI = [-0.04, 0.29]. Chinese VS participants were more influenced by the verb, *M* = 0.54, 95% CI = [0.39, 0.69], than by the adverb, *M* = 0.13, 95% CI = [0.04, 0.22]. Likewise, Chinese VP participants were more influenced by the verb, *M* = 0.61, 95% CI = [0.45, 0.77], but relative to the other FFI groups, maintained some sensitivity toward the adverb cue, *M* = 0.47, 95% CI = [0.30, 0.64]. The one VP participant who attained less than 88% in Phase 1 pretraining showed little influence of verb bias in later comprehension *(r* = 0.11) compared to adverb bias (*r* = 0.88).

An ANOVA (4 Groups × 2 Cues, with subjects nested within groups) revealed an overall effect of group, *F*(3,63) = 3.83, *p* = 0.01; and a significant group by cue interaction, *F*(3,63) = 7.80, *p* < 0.001. Individual ANOVAs (2 Groups × 2 Cues) of each FFI group against the CC were conducted using Bonferroni adjusted alpha levels of 0.017 per test (0.05/3). The results yielded a significant interaction of group and cue for the CC group versus the VG group, *F*(1,35) = 18.73, *p* < 0.001; for the CC group versus the VS group, *F*(1,32) = 25.84, *p* < 0.001; and for the CC group versus the VP group, *F*(1,32) = 8.51, *p* = 0.006. All FFI treatments therefore increased sensitivity to the verb cue.

**FIGURE 4:**
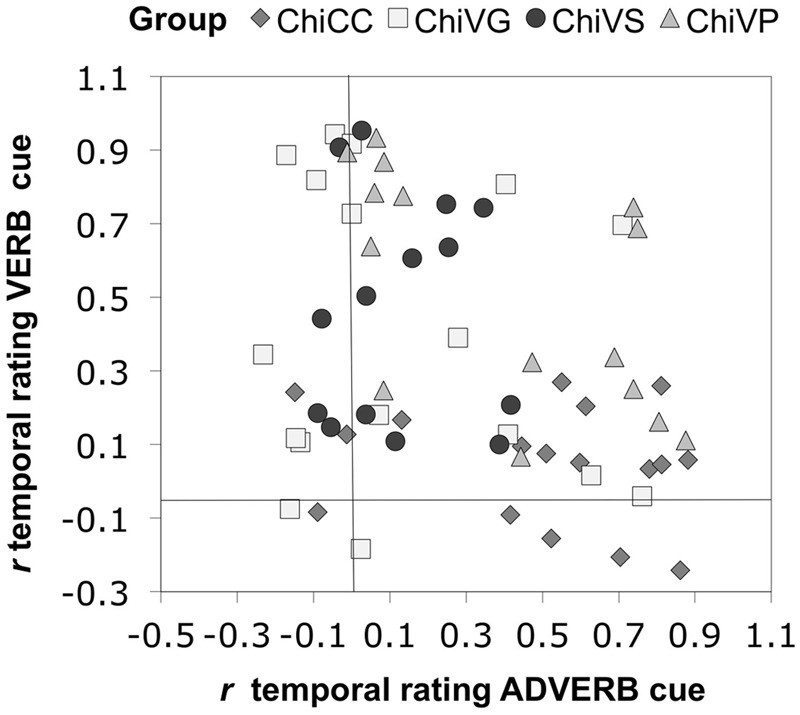
**Sensitivity to adverbial and verbal inflectional cues to temporal reference in each participant.** Chi = Chinese.

**Figure 4** shows the reliability of these patterns across individual group members. Most CC individuals were predominantly influenced by the adverb cue, whereas most VS and VG participants were more influenced by the verb cue. Verb pretraining participants were more scattered: most showed greater sensitivity to the verb, though there were some who lay close to the 45° diagonal, suggesting that they were more evenly affected by both cues.

### Eye-Tracking Data

#### Sentence Exposure

**FIGURE 5:**
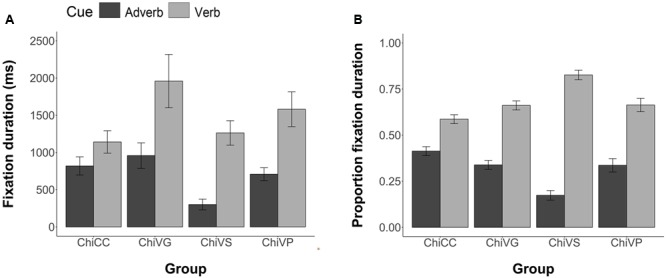
**Mean Group Fixation Duration on the Adverb and Verb cues in Sentence Exposure. (A)** Participants’ total fixation duration during Exposure. **(B)** Participants’ proportion fixation duration during exposure. Error bars are 2 standard errors long. Chi = Chinese.

**Figure 5** and **Table 2** show the group mean fixation duration of these participants as they were studying the adverb and verb cues during exposure to the Latin sentences. **Figure 5A** shows the total fixation duration on these cues. **Figure 5B** shows these data as the proportion of the total fixations on each trial. The pattern in these Figures is clear, all groups looked at the verb more than the adverb, but it was the three FFI groups that did so to a greater extent. Individual ANOVAs on the total fixations (2 Groups × 2 Cues, with subjects nested within groups) were conducted using Bonferroni adjusted alpha levels of 0.017 per test (0.05/3). The results revealed a significant group by cue interactions for the VG group versus the CC group, *F*(1,29) = 6.86, *p* = 0.014; for the VS group versus the CC group, *F*(1,28) = 17.71, *p* < 0.001; but the interaction marginally failed to reach significance for the VP group versus the CC group, *F*(1,28) = 4.06, *p* = 0.05. VG and VS therefore paid more attention than the CC group to the verb cue during processing.

**Table 2:** Mean participant fixations on the adverb and verb cues by the four groups of Experiment 1.

Group	Cue	Mean	95% CI
**Mean total fixation duration (ms.)**			
Control	Adverb	819.2	[402, 1236]
	Verb	1141.6	[628, 1656]
Verb grammar	Adverb	958.7	[340, 1577]
	Verb	1959.2	[662, 3256]
Verb salience	Adverb	300.3	[23, 578]
	Verb	1262.7	[631, 1894]
Verb pretraining	Adverb	708.8	[375, 1041]
	Verb	1581.1	[681, 2480]
**Mean proportion fixation time**			
Control	Adverb	0.41	[0.33, 0.50]
	Verb	0.59	[0.50, 0.67]
Verb grammar	Adverb	0.34	[0.25, 0.43]
	Verb	0.66	[0.58, 0.75]
Verb salience	Adverb	0.17	[0.07, 0.27]
	Verb	0.83	[0.73, 0.93]
Verb pretraining	Adverb	0.34	[0.20, 0.48]
	Verb	0.66	[0.52, 0.80]

#### Correlations between Attention to Cue in Sentence Exposure and Subsequent Cue Comprehension

Pearson correlations investigating the relations between attention in the Sentence Exposure phase and comprehension ability in the Comprehension Phase across all the participants and groups of Experiment 1 show that the proportion of fixation time spent on the adverb during Sentence Exposure correlates significantly with later adverbial bias in Comprehension (*r* = 0.50, *p* < 0.001). Likewise, proportion of fixation time spent on the verb during Sentence Exposure correlates significantly with later verb bias in Comprehension (*r* = 0.45, *p* < 0.001).

#### Sentence Exposure Eye-Tracking Over Trials

**FIGURE 6:**
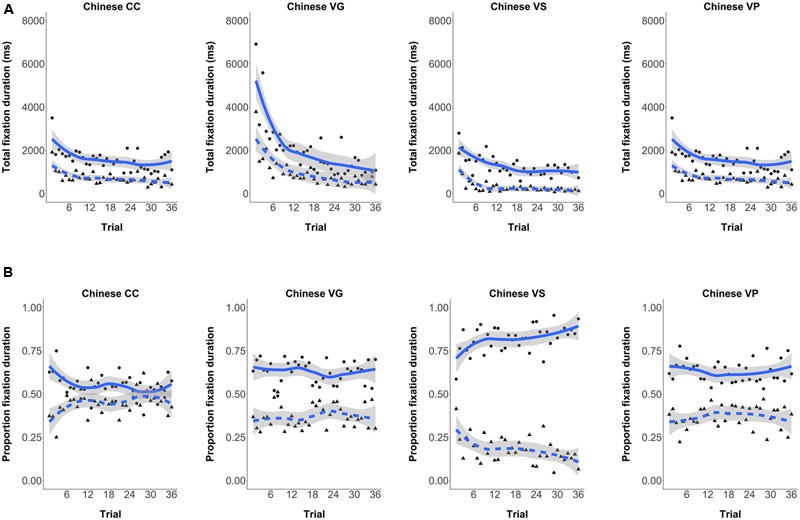
**Mean Group Fixation Duration on the Adverb and Verb cues Over Trials (solid lines and circles = verb, and dotted lines and triangles = adverb). (A)** Participants’ total fixation duration by each trial of exposure. **(B)** Participants’ proportion fixation by each trial of exposure.

Although the random order of stimuli was different for each participant, we can determine the degree to which the participants attended to the verb and adverb cues over trials. **Figure 6A** shows the total fixation on each cue by trial of experience in all L1 Chinese groups. It can be seen that CC participants initially spent more time looking at the verb, but interest in this cue waned over trials and more attention was paid to the adverbial cue. Participants in the three FFI conditions, however, maintained a steady attentional preference for the verb cue. These patterns are clearer in **Figure 6B**, which plots the proportion of fixation time on each trial spent on the adverb and verb cues, respectively.

## Discussion

The behavioral results of Experiment 1 show that under CC, adverbs were better attended than verb inflections. This finding replicates that of Ellis and Sagarra (2010, 2011) and Cintrón-Valentín and Ellis (2015). In the linguistic input, adverbial cues are more salient, simple and reliable cues compared to the verb-tense inflections. Furthermore, the adult language learners’ prior use of adverb temporal reference in their Chinese L1 could have resulted in long-term blocking. In contrast to the CC treatment, training on the isolated verb cue under the VP condition reversed this bias, resulting in a better use of the verb cue during comprehension. This finding also replicates that of Ellis and Sagarra (2010, 2011) and Cintrón-Valentín and Ellis (2015), showing short-term learned attention effects, where prior learning of an isolated cue during pretraining shifts learners’ attention to that cue in subsequent testing. In the two other FFI conditions, VG, where learners were first exposed to a short instructional sequence on how Latin verb-tense morphology works in Latin, and VS, where the verb inflections were made more salient by means of textual enhancement manipulations during exposure, participants were better able to use the verb cues in comprehension relative to the adverb cue than those in the CC condition. Of the three FFI conditions, VP resulted in more balanced acquisition of both verbal and adverbial cues.

The eye-tracking data show how these FFI treatments affected attention to cues in the input processing. All participants looked at the verbs more than they did the adverb during sentence exposure. However, participants in the VG and VS conditions fixated upon the verbs significantly more during input processing than did Control participants. The VP group, however, did not differ significantly from the control group, although the same numerical trend was evident. The correlation analyses suggest that the relative amount participants spent processing the verb/adverb cues during exposure determined cue usage in subsequent comprehension testing. The trial-by-trial analyses illustrated in **Figure 6**, show that CC participants initially spend more time looking at the verb, however, participants rapidly lose interest in the verb cue across trials and more attention is paid to the adverbial cue. One possible interpretation is that learners initially first fixate more on the verb + inflection because it is the longer word form, however, over trials they come to realize that the adverb is the simpler and more reliable cue, and as a result they shift their attention to it. The FFI participants on the other hand – for whom the verb forms or their functions were made more salient – pay more attention to verb from the start of language exposure, and this focus persists, leading to subsequent attention and use of this cue.

## Experiment 2

### Introduction

Our previous studies examining the effects of learned attention and blocking and the effects of FFI in overcoming learned attentional biases in L2 acquisition Ellis and Sagarra (2010, 2011) and Ellis et al. (2014) have focused on the learning of Latin only through the visual modality. As described in the introduction, spoken and written language are very different mediums. Whereas readers have the advantage of being able to control the amount and speed at which they process visual input, the fleeting nature of spoken language does not afford listeners the same advantage. These differences could well-affect the degree to which different language forms are salient in the input and thus control the degree to which they are attended, perceived, processed, and learned. Indeed Leow (2015, p. 122), in reviewing the relevance for instruction of this work on learned attention, explicitly asks for a potential replication study which addresses the issue of whether the findings can be extrapolated to the aural mode. This experiment therefore aims to replicate and extend previous work by comparing the attentional processes of L1 speakers of English in control (CC), VG, VS, and VP conditions who learn from aural input with those whose input experience is visual.

### Participants

Participants were 200 individuals recruited from a major university in the USA. They were volunteers who participated as part of an undergraduate psychology course requirement (*n* = 182) or were paid $10 for their participation (*n* = 18). Inclusion criteria required participants to be native English speakers who had not learned Latin or Italian previously. They could know Spanish but could not have been raised bilingually before the age of 6 years. They were randomly assigned to one of eight conditions regarding instruction and modality of presentation. Those who received Aural presentation only were split into CCA (Control Condition Aural), *n* = 25 (15 females and 10 males), age range 17–45 years (*M* = 21.56); VGA (Verb Grammar Aural), *n* = 25 (16 females and 9 males), age range 18–22 years (*M* = 18.84); VSA (Verb Salience Aural), *n* = 25 (22 females and 3 males), age range 17–20 years (*M* = 18.36); and VPA (Verb Pretraining Aural), *n* = 25 (15 females and 10 males), age range 18–20 years (*M* = 18.44). Participants who received instruction in the Visual modality only were split into CCV, *n* = 25 (18 females and 7 males), age range 18–21 years (*M* = 18.40); VGV (Verb Grammar Visual), *n* = 25 (8 females and 17 males), age range 17–22 years (*M* = 18.68); VSV (Verb Salience Visual), *n* = 25 (9 females and 16 males), age range 18–20 years (*M* = 18.68); and VPV (Verb Pretraining Visual), *n* = 25 (14 females and 11 males), age range 18–21 years (*M* = 18.52).

### Procedure

The experiment was programmed in *PsychoPy* (Peirce, 2007) and consisted of the same phases as presented in Experiment 1 (see **Table 3** for detailed procedure). However, the stimulus set for Experiment 2 was more complex than that of Experiment 1. In Experiment 1 participants were presented with one verb stem, *cogit-*, which was combined with all appropriate past, present and future inflections, whereas in Experiment 2, participants were presented with four different verb stems and their appropriate past, present and future inflections.

**Table 3:** The design of Phases 1–3 of Experiment 2.

Pretraining (Phase 1) (+ feedback)	Sentence Exposure (Phase 2) (+ feedback) 48 (24 × 2) randomized trials	Comprehension Test (Phase 3) (- feedback) 48 randomized trials	
		Stimulus	Semidiem
**Control group**	**Present**		
No pretraining **Verb Pretraining group** (36 randomized trials) *nato* “I swim” *cantas* “You sing” *pugnas* “You fight” *fleat* “He/She cries” *cantavi* “I sang” *natavisti* “You swam” *pugnavit* “He/She fought” *fleavit* “He/She cried” **Verb Salience group** No pretraining **Verb Grammar group** Brief Grammar Lesson See **Figure 1**	*hodie cantas* *hodie fleat* *hodie pugnas* *hodie nato* *cantas hodie* *fleat hodie* *pugnas hodie* *nato hodie* **Past** *heri cantavi* *heri fleavi* *heri fleavit* *heri pugnavit* *heri natavisti* *cantavi heri* *fleavi heri* *fleavit heri* *pugnavit heri* *natavisti heri* **Future** *cras cantabit* *cras pugnabis* *cras natabo* *cantabit cras* *pugnabis cras* *natabo cras*	*verb-o/as/at cras* *verb-avi/visti/vit cras* *verb-abo/ bis/ bit cras* *hodie verb–o/as/at* *hodie verb-avi/visti/vit* *hodie verb-abo/bis/bit* *heri verb-o/as/at* *heri verb-avi/visti/vit* *heri verb-abo/bis/bit* *cras verb-o/as/at* *cras verb-avi/visti/vit* *cras verb-abo/bis/bit* *verb-o/as/at hodie* *verb-avi/visti/vit hodie* *verb-abo/bis/bit hodie* *verb-o/as/at heri* *verb-avi/visti/vit heri* *verb-abo/bis/ bit heri*	3 1 5 3 2 4 2 1 3 4 3 5 3 2 4 2 1 3

*The rating scale for the Comprehension Test ranged from 1 (past) to 5 (future). The correct answer for each trial is shown in the semidiem column.*

#### Pretraining

**FIGURE 7:**
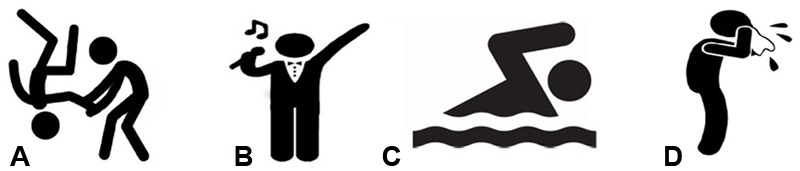
**Pictures presented during Verb Pretraining, Exposure and Comprehension testing.** During Pretraining and Exposure each picture was presented in the presence of the corresponding Latin verb (either aurally or visually, depending on the condition), where **(A)** corresponded to any form of the verb *pugno* (representing the verb *to* fight in English); **(B)** corresponded to any form of the verb *canto* (representing the verb *to* sing in English); **(C)** corresponded to any form of the verb *nato* (representing the verb *to* swim in English); and **(D)** corresponded to any form of *fleo* (representing the verb *to* cry in English). During the Comprehension testing phase, a pane where all four pictures were present was shown in each trial, again in the presence of the corresponding Latin verb.

Participants in the VP group were first pretrained on verb inflections and determined that each made reference to either present or past time. On each trial, Visual participants saw, or Aural participants heard, one of the four verb stems (*cant-, flea-, nat-, pugn-*) combining an inflection referencing the past (*-avi* -*avisti, -avit)* or present (*-o, -as, -at*). Participants were additionally presented with a picture of a stick figure that represented the action of the verb (see **Figure 7**). They were asked to select the Latin verb’s temporal (past/present) reference from an on-screen menu. Feedback was provided on their responses. In this phase they were not asked about the verb meaning, thus their understanding was focused upon the morphological tense reference.

Pretraining for the VG participants involved a brief lesson on Latin verb-tense morphology using similar slides to those shown in **Figure 1**, except that the Latin verb *amare* was used as an example in slides 2 and 3. Regardless of modality of language exposure these slides were presented visually.

#### Sentence Exposure

In Sentence Exposure, Visual participants saw, Aural participants heard, 24 different sentence combinations, which appropriately combined the adverb with a verb stem (see **Table 3**). While the sentence was exposed, participants saw onscreen a picture of a stick figure which appropriately represented the action the verb was referencing. Again, in this phase they were not asked to make any judgments regarding the picture they were shown. After each sentence, participants were asked to identify whether the sentences referred to the past, present, or future, responding via a visual menu presented on the computer screen. The sample of stimuli selected for presentation in Sentence Exposure ensured that each verb root was (1) presented once in each tense, and (2) appropriately combined one of the agreement markers for each tense. The Sentence Exposure procedure was identical for the CC, VG, and VP groups. For VS participants only, the stimuli were either textually or aurally enhanced to increase their salience, so for Visual presentation the verbal inflections were highlighted in bold and red, and for Aural presentation the verb inflections were spoken emphatically. Feedback was given for both correct and incorrect choices. For correct answers, the word “correct” would appear on the screen, whereas for incorrect answers, participants would see the word “wrong” accompanied by the correct answer (e.g., “Wrong – [*heri cantavit*] is [past]”).

#### Comprehension Test

Here, participants were presented with randomized verb-adverb/adverb-verb combinations as well as a selection of single word items. The single word items were verbs (*canto, fleat, natat, pugnas, cantavi, fleavit, natavit, pugnavisti, cantabit, fleabis, natabo, pugnabit*), half of which had been previously presented in the same inflection during Sentence Exposure. For the randomized verb-adverb/adverb-verb combinations, similar to Experiment 1, participants experienced sentences that were familiar to them from the previous task, but also combinations in which the verb and adverb were incongruent in their time reference. Here, participants saw six congruent combinations they had previously experienced during Sentence Exposure (*heri fleavi, heri pugnavit, heri natavisti, hodie cantas, hodie nato, cras pugnabis*) as well as six new congruent combinations they had not seen before (*heri natavi, heri cantavit, hodie fleo, hodie fleas, cras cantabis, cras pugnabo*). For the trials involving incongruent combinations, each of the verbs used for the congruent combinations were combined with all possible adverb forms. Overall this led to a total of 12 single-word items and 36 two-word combinations. In each of the trials, participants were additionally presented with a four-picture pane menu, where they saw the four pictures they had been previously presented with during Sentence Exposure. The position of the pictures was counterbalanced in the pane.

On each trial, participants were asked to make two judgments. The first judgment was whether the word string referred to the past, present, or future on a five-point scale. The possible scale points were the same as in Experiment 1. For the second judgment, participants were asked to select the picture that best represented the word or phrase they were presented with. This judgment tested how well they had processed the meaning of the verbs to which they had been exposed. Feedback was not provided.

## Results

### Visual Modality

#### Verb Pretraining Data

Mean performance in the first quarter of Verb Pretraining was 63%. By the fourth quarter, mean performance was 86%, demonstrating acceptable completion of Phase 1.

#### Sentence Exposure Data

Mean performance in the first quarter of Sentence Exposure was 61% for the CCV group, 84% for the VGV group, 68% for the VSV group, and 82% for the VPV group. Both the VGV and the VPV groups were at an advantage in the first quarter compared to the other groups. However, performance evened out for all groups by the end of the phase. Mean performance in the final quarter was 96% for the CCV group, 98% for the VGV group, 93% for the VSV group, and 95% for the VPV group. A one-way ANOVA on these final quarter scores did not reveal a significant group effect, *F*(3,96) = 1.71, *p* = 0.17.

#### Comprehension Data

##### Perception of Time Cues

**FIGURE 8:**
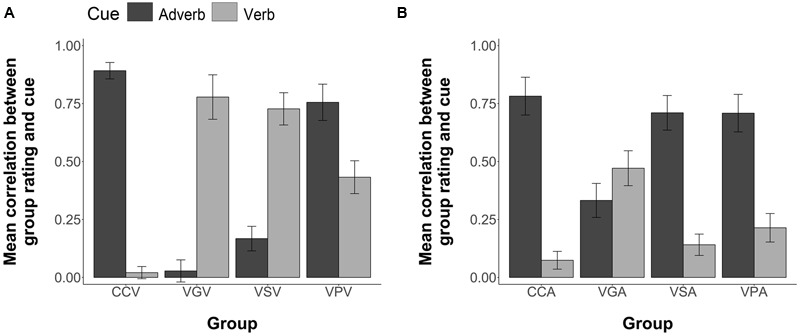
**Group mean correlations between individual participants’ Comprehension sentence ratings and the information given by the corresponding adverb and verb cues. (A)** Group mean correlations for the visual modality treatments **(B)** Group mean correlations for the aural modality treatments. Error bars are 2 standard errors long.

Each participant’s temporal rating responses for the strings in Comprehension testing were correlated with the information provided by the verb cue and the information separately provided by the adverb cue to determine the degree to which each participant was biased by each cue type. Pearson correlations between each participant’s temporal rating responses and the information provided by the verb and adverb cues separately are illustrated in **Figure 8A**. CCV participants were more influenced by the adverb, *M* = 0.89, 95% CI = [0.82, 0.96] than the verb, *M* = 0.02, 95% CI = [-0.03, 0.07]. VGV participants were more influenced by the verb, *M* = 0.78, 95% CI = [0.59, 0.97], than by the adverb, *M* = 0.03, 95% CI = [-0.07, 0.12]. VSV participants were more influenced by the verb, *M* = 0.73, 95% CI = [0.59, 0.86], than by the adverb, *M* = 0.17, 95% CI = [0.06, 0.27]. Contrary to the other FFI conditions VPV participants were more influenced by the adverb, *M* = 0.81, 95% CI = [0.60, 0.91], but showed some sensitivity toward the verb cue, *M* = 0.43, 95% CI = [0.29, 0.57].

If we compare the comprehension data for the VPV participants with that of the Chinese VP participants in Experiment 1 (verb: *M* = 0.61, 95% CI = [0.45, 0.77]; adverb: *M* = 0.47, 95% CI = [0.30, 0.64], the pattern is quite different, with the VPV participants showing a greater degree of sensitivity toward the adverb cue relative to the verb cue. Although the VPV participants showed an increase in their sensitivity toward the verb cue when, compared to the CCV group (as confirmed by our analysis of variance below), it seems that the greater complexity of the stimulus set in Experiment 2 had an impact on the learners’ attentional focus, and thus on the degree of sensitivity they showed toward the verbal morphological cues during comprehension.

An ANOVA (4 Groups × 2 Cues, with subjects nested within groups) revealed a significant group by cue interaction, *F*(3,96) = 7.80, *p* < 0.001. As in Experiment 1, individual ANOVAs (2 Groups × 2 Cues) of each FFI group against the CC were conducted using Bonferroni adjusted alpha levels of 0.017 per test (0.05/3). The results yielded a significant interaction of group and cue for the CCV group versus the VGV group, *F*(1,48) = 82.19, *p* < 0.001; for the CCV group versus the VSV group, *F*(1,48) = 73.72, *p* < 0.001; and for the CCV group versus the VPV group, *F*(1,48) = 8.68, *p* = 0.004. These results replicate those of Experiment 1, where the FFI conditions increased sensitivity to the verb cue.

**FIGURE 9:**
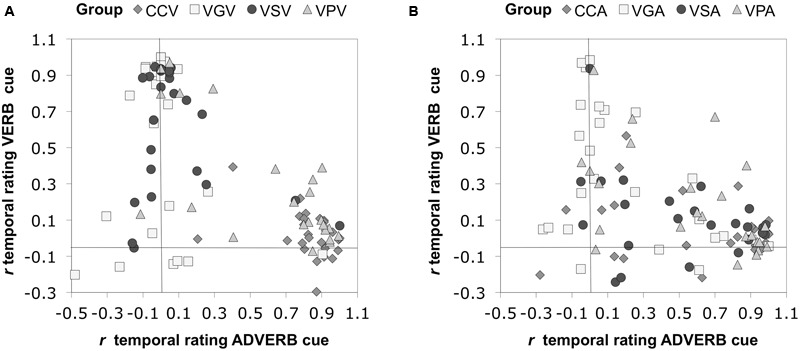
**Sensitivity to adverbial and verbal inflectional cues to temporal reference in each participant. (A)** The groups in the visual modality. **(B)** The groups in the aural modality.

**Figure 9A** shows the reliability of these patterns across individual group members. For the groups in the visual modality, most CCV participants were influenced by the adverb cue, whereas most VSV and VGV participants were more influenced by the verb cue. VPV participants were more scattered: most showed greater sensitivity to the adverb, though there were some who showed greater sensitivity toward the verb, and one participant who lay close to the 45° diagonal, suggesting that they were more evenly affected by both cues.

**FIGURE 10:**
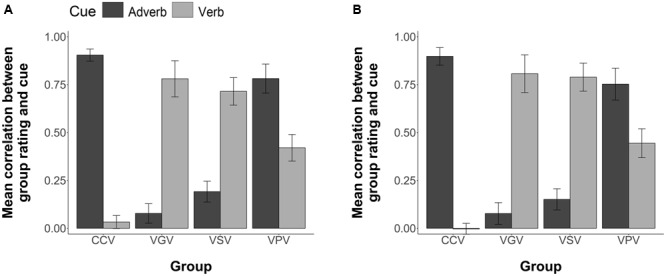
**Group mean correlations between individual participants’ Comprehension sentence ratings and the information given by the corresponding adverb and verb cues for the Visual Modality. (A)** Group mean correlations for the trained items **(B)** Group mean correlations for the generalization items. Error bars are 2 standard errors long.

To determine if the effects of FFI on cue use during comprehension testing differed based upon the nature of the items, that is, whether they were trained (i.e., previously presented during Sentence Exposure) or generalization items (i.e., only presented during Comprehension Testing) we ran a three-way ANOVA (4 Groups × 2 item type × 2 cues). The analyses revealed a non-significant effect of item type *F*(1,96) = 0.19, *n.s.*, a statistically significant group by cue interaction, *F*(3,96) = 28.56, *p* < 0.001, but no significant three-way interaction between item type (Trained or Generalization), group, and cue use, *F*(3,96) = 0.69, *p* = 0.56. Thus, participants performed at a similar level, regardless of item type (see **Figure 10**).

##### Perception of Verb Meaning

Mean accuracy scores for verb meaning was 0.82 for the CCV group, 0.80 for the VGV group, 0.76 for the VSV group, and 0.91 for the VPV group. A one-way ANOVA on each of the conditions’ mean accuracy scores for the picture ratings showed a non-significant effect of group, *F*(3,96) = 2.59, *p* = 0.06. *Post hoc* Tukey HSD tests demonstrated just one significant pairwise group difference: between the VPV group, and the VSV group, *p* = 0.04, 95% CI = [0.004, 0.30].

### Aural Modality

#### Verb Pretraining Data

Mean performance in the first quarter of Verb Pretraining was 66%. By the fourth quarter, mean performance was 80%, demonstrating acceptable completion of Phase 1.

#### Sentence Exposure Data

Mean performance in the first quarter of Sentence Exposure was 49% for the CCA group, 50% for the VGA group, 54% for the VSA group, and 67% for the VPA group. The pretraining on the verb allowed the VPA participants to be at an advantage in the first quarter compared to the other groups, and this advantage also persisted. Mean performance in the final quarter was 78% for the CCA group, 68% for the VGA group, 77% for the VSA group, and 87% for the VPA group. A one-way ANOVA on these final quarter scores revealed a significant group effect, *F*(3,96) = 3.12, *p* = 0.03. *Post hoc* Tukey HSD tests demonstrated one significant pairwise group difference: between the VPA group, and the VGA group, *p* = 0.02, 95% CI = [0.02, 0.35].

#### Comprehension Data

##### Perception of Time Cues

Pearson correlations between each participant’s temporal rating responses and the information provided by the verb and adverb cues separately are illustrated in **Figure 8B**. CCA participants were more influenced by the adverb, *M* = 0.78, 95% CI = [0.62, 0.94] than the verb, *M* = 0.07, 95% CI = [-0.001, 0.15]. VGA participants were more influenced by the verb, *M* = 0.47, 95% CI = [0.32, 0.62], than by the adverb, *M* = 0.33, 95% CI = [0.19, 0.48]. Contrary to the VGA condition, VSA participants were more influenced by the adverb, *M* = 0.71, 95% CI = [0.56, 0.86], than by the verb, *M* = 0.14, 95% CI = [0.05, 0.23]. Likewise, VPA participants were more influenced by the adverb, *M* = 0.71, 95% CI = [0.55, 0.99], but showed some sensitivity toward the verb cue, *M* = 0.21, 95% CI = [0.09, 0.33].

The general patterns observed here were reliable across individual group members. **Figure 9B** shows the aural modality data. Again, most CCA individuals were predominantly influenced by the adverb cue. VGA participants showed greater sensitivity toward the verb, whereas VSA participants were predominantly influenced by the adverb. Similar to those in the visual modality, VPA participants were more scattered: most showed greater sensitivity toward the adverb, a small group of participants showed greater sensitivity toward the verb, and one participant lay close to the 45° diagonal, suggesting that they were more evenly affected by both cues.

An ANOVA (4 Groups × 2 Cues, with subjects nested within groups) revealed a significant group by cue interaction, *F*(3,96) = 5.53, *p* = 0.002. Individual ANOVAs (2 Groups × 2 Cues) of each FFI group against the CCA group yielded a significant interaction of group and cue for the CCA group versus the VGA group, *F*(1,48) = 13.09, *p* < 0.001; a significant main effect of cue for the CCA group versus the VSA group, *F*(1,48) = 35.32, *p* < 0.001, but no significant group by cue interaction, *F*(1,48) = 0.63, *p* = 0.43; and a significant main effect of cue for the CCA group versus the VPA group, *F*(1,48) = 30.15, *p* < 0.001, but no significant group by cue interaction, *F*(1,48) = 1.07, *p* = 0.31. Contrary to the results of Experiment 1 and those for Visual presentation described in 3.4.1, it seems only the VGA group increased sensitivity to the verb cue.

**FIGURE 11:**
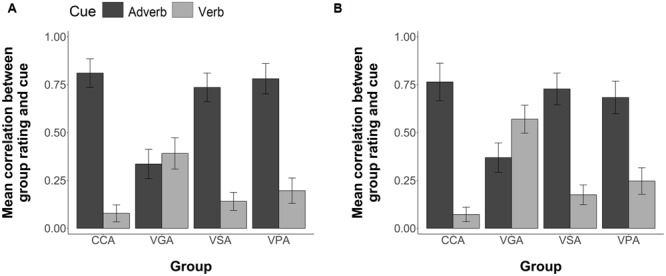
**Group mean correlations between individual participants’ Comprehension sentence ratings and the information given by the corresponding adverb and verb cues for the Aural Modality. (A)** Group mean correlations for the trained items **(B)** Group mean correlations for the generalization items. Error bars are 2 standard errors long.

As for the visual modality, to determine if the effects of FFI on cue use during comprehension testing differed based upon the nature of the items (i.e., Trained or Generalization) we ran a three-way ANOVA (4 Groups × 2 item type × 2 cues). The analyses revealed a non-significant effect of item type *F*(1,96) = 0.0002, *n.s*., a statistically significant item type by cue interaction, *F*(1,96) = 8.04, *p* = 0.006, but no significant three-way interaction between item type (Trained or Generalization), group, and cue use, *F*(3,96) = 0.70, *p* = 0.55. Thus, similar to the visual modality, participants in the aural modality performed at a similar level, regardless of item type (see **Figure 11**).

##### Perception of Verb Meaning

Mean accuracy scores for verb meaning was 0.59 for the CCA group, 0.56 for the VGA group, 0.63 for the VSA group, and 0.80 for the VPA group. A one-way ANOVA on each of the conditions’ mean accuracy scores for the picture ratings showed a significant effect of group, *F*(3,96) = 5.35, *p* = 0.002. *Post hoc* Tukey HSD tests demonstrated three significant pairwise group differences: between the VPA group and the CCA group, *p* = 0.01; between the VPA group and the VGA group, *p* = 0.002; and between the VPA group and the VSA group, *M* = 0.62, *p* = 0.05.

### Modality by FFI Interactions

To determine if the effects of FFI on cue use during comprehension testing differed across modality of presentation, we ran a three-way ANOVA (4 Groups × 2 modalities × 2 cues). The analyses revealed a statistically significant three-way interaction between modality of input presentation (Aural or Visual), group, and cue use, *F*(3,384) = 9.85, *p* < 0.001.

Inspection of **Figure 8** shows the major loci of this interaction. Under CC, participants process the adverb and pay little or no heed to the verb. This is so for CCA and CCV. Pretraining on the verb in VP allows them a little better use of this cue, especially in VPV, but still it is overshadowed by the adverb. Making the verbal inflections salient during sentence exposure with visual presentation VSV allows learners to attend and learn to use verb morphology. But this is absolutely not so with auditory presentation VSA. Grammar instruction, however, does allow learners to make use of the morphological cues, both from auditory presentation, and particularly from visual exposure.

## Discussion

The findings for the Visual conditions follow that of the prior learned attention studies. The CCV group showed greater sensitivity toward the adverb than the verb cue. The VPV treatment allows participants to show sensitivity toward the verb cue, while also showing sensitivity to use of adverbs. As in Experiment 1, and in Cintrón-Valentín and Ellis (2015), both VG and VS treatments in the visual modality shifted learners’ attention to the verb cue in subsequent testing.

The general behavioral data for the Aural conditions show consistency in that learners in the CCA group focus more on the adverb than the verb cues. However, the other findings are in contrast to these patterns following Visual exposure. Of the three FFI conditions, only VGA produced a shift in their attention toward the verb cue. VSA participants’ performance was similar to that of the CCA participants, showing more sensitivity toward the adverb relative to the verb cue. Although VPA participants showed an increase in their verb sensitivity, when compared to that of the CCA group, their attention was greater toward the adverb cue than to the verb cue. We will discuss these disparities below.

## General Discussion

Experiment 1 tested whether under normal conditions of exposure (CC), the effects of physical salience and learned attentional biases toward adverbial cues, prejudice the acquisition of verbal tense morphology, as indexed in participants’ relative reliance on these cues in subsequent language comprehension (RQ1). The results of Experiment 1 lend support to the idea that the limited attainment of adult second and foreign language learners follows general principles of associative learning and cognition wherein salience and attention are key factors (Ellis, 2006a,b). Under normal conditions of language exposure (CC), adverbs were better processed than verb inflections. We interpret this phenomenon, a standard finding in SLA research (e.g., Schmidt, 1984; Bardovi-Harlig, 1992, 2000; Noyau et al., 1995; Van Patten, 1996, 2006; Clahsen and Felser, 2006), as relating firstly to the relative salience, simplicity and reliability of adverb cues which render them more learnable when compared to verb-tense morphology, and secondly, to adult language learners’ prior knowledge of the use of adverb temporal reference in their L1 which results in the long-term blocking of these forms. This is apparent in adult language learners’ difficulty in learning morphology compared to child learners, and in studies such as Ellis and Sagarra (2010, Experiment 2) and Ellis and Sagarra (2011, Experiments 2 and 3) which demonstrate long-term language transfer effects whereby the nature of learners’ first language (+/- verb tense morphology) biased the acquisition of morphological versus lexical cues to temporal reference in Latin. First language speakers of Chinese (no tense morphology) were less able than first language speakers of Spanish or Russian (rich morphology) to acquire inflectional cues from the same language experience.

Experiment 1 also tested whether early experience of morpho-logical cues to temporal reference, through each of the FFI treatments VG, VS, and VP, counteract the effects of physical salience and learned attentional biases, as indexed by participants’ relative reliance on these cues in subsequent language comprehension (RQ2). The behavioral data demonstrated that all FFI interventions resulted in better attention to and use of the verbal inflectional cues. Participants in the VG group were initially provided with declarative statements about morphological function but still had to put this knowledge to use in subsequent phases. VS learners were introduced to the verbal cues during the exposure phase but still had to determine their function. Both of these treatments resulted in participants’ attending these cues and using them over adverbial cues. In contrast, the verb pretraining in VP, where learners had to process the Latin verb forms for meaning in English, resulted in a more balanced acquisition of both verbal and adverbial cues. We believe that this is because, having learned to some extent how to use the morphology, they were next able to consider the role of adverbs too. This interpretation is consonant with other findings in the literature, where in the early stages of learning, when learners are confronted with multiple cues to interpretation, they typically focus upon one cue at a time. As they reduce errors of estimation regarding the outcome or interpretation of the cue, they then consider the role of the other cues (MacWhinney, 2001).

The results of the VG condition are consonant with prior findings in the literature on FFI (see, for instance, the meta-analyses of effects of type of instruction by Norris and Ortega, 2000; Spada and Tomita, 2010; Goo et al., 2015), which suggest that instructional conditions involving a focus on the rules underlying specific L2 structures generally lead to large advantages in the acquisition of target forms. In terms of the results obtained for the VS condition, as we explained in the introduction, reviews of the effectiveness of textual enhancement (TE) have yielded conflicting findings (Han et al., 2008; Lee and Huang, 2008), largely due to a wide variety of methodological differences. However, one pattern that seems to apply is that the provision of compound enhancement, that is, “TE in combination with attention-getting strategies such as corrective feedback” (Han et al., 2008, p. 609) tends to be more effective in encouraging noticing and subsequent processing than simple enhancement. The sentence exposure phase for our VS participants involved exactly this – visual salience and corrective feedback.

Two additional research questions in Experiment 1 concerned (i) whether early experience of morphological cues to temporal reference lead to biases in subsequent overt perceptual attention (as indexed by number of fixations) during Sentence Exposure, where there are both adverbial and morphological cues to the same interpretation (RQ3), and (ii) whether any bias in overt attention to input cues in turn lead to subsequent attentional biases to the adverbial or morphological cues in subsequent language comprehension (RQ4). The eye-tracking data from Experiments 1 show how the FFI treatments affected attention to cues during input processing. All participants fixated upon the verbs significantly more than they did the adverb. As shown by the group by cue interactions, participants in the VG and VS conditions attended the inflections more than the control participants, as did the VP participants although in the latter case the interaction failed to reach significance. Additionally, the correlation analyses showed that the relative amount participants spent processing the verb/adverb cues during language exposure determined their cue usage in subsequent comprehension.

The trial-by-trial analyses of **Figure 6** show that although control participants initially spent more time looking at the verb, their interest in this cue waned over trials, with more attention being paid to the adverbial cue. One interpretation is that CC learners initially first fixated more on the verb + inflection because it is the longer more salient form. Initially, over the first 20 trials or so, they tried to induce the system of how the inflections signal temporality, but realizing that the adverb is the simpler and more reliable cue, they eventually shifted their attention to it. The FFI groups on the other hand – who were initially made aware of the verb forms or their functions, (1) by having the verb-inflections explained during pretraining (VG), (2) by having the inflections made more salient by textual enhancement during exposure (VS), (3) or by being pretrained on the verbal cues in non-redundant situations (VP) – paid more attention to the verbs from the start of the exposure phase, and this persisted through the end of the trials. Overall, the eye movement findings in the current study replicate with Chinese L1 speakers those of Cintrón-Valentín and Ellis (2015) with English L1 learners.

Experiment 2 investigated the effects of modality of language exposure upon the salience and consequent processing of linguistic form, and on the ways in which different types of FFI interact with these very different mediums. The fleeting nature of spoken language does not afford listeners the control of scrutiny of input as does visual presentation, and these differences could well-affect the degree to which forms are salient in the input. This experiment therefore compared the attentional processes of L1 speakers of English in control (CC), VG, VS, and VP conditions who were exposed to aural input with those whose input experience was visual. Consonant with prior studies, it showed that participants under control conditions (either CCV or CCA) showed greater sensitivity toward the adverb than the verb cue.

Two specific research questions in Experiment 2 concerned (i) whether each of the FFI treatments VG, VS, and VP, counteract the effects of physical salience and learned attentional biases, as indexed by participants’ relative reliance on these cues in subsequent language comprehension (RQ5), and (ii) whether each of the FFI treatments VG, VS, and VP are equally effective in reattuning learners’ attention to the non-salient morphological cues through visual and auditory modalities of exposure (RQ6). Regarding RQ5, all forms of FFI were effective in increasing attention to verbal morphology in the visual modality, although VP resulted in balanced attention to both cues. However, it was generally the case that attending to the morphological cues was considerably more difficult under aural than under visual presentation. Only grammar instruction (VG) was successful in reattuning learners’ attention to the non-salient morphological cues in both modalities. This instruction allowed learners to become aware of forms and their patterning of function prior to sentence exposure, and their subsequent processing of these cues in the input promoted their use during comprehension testing. Also relating to RQ6, a major difference was seen in the effectiveness of morphological salience-raising in the two modalities. Increasing the salience of the verb morphology was very effective with visual exposure. Textually enhancing the morphology promoted attention to and analysis of these cues during sentence exposure, and this in turn resulted in subsequent use of these cues even when no longer made salient. In contrast, although emphasized pronunciation of these cues led to their use during sentence exposure in the aural group, this did not result in sustained use of these cues once the emphasis was removed. With aural presentation, the emphasized inflectional forms were attended but not analyzed, so that abrupt removal of the emphasis removed the cues themselves.

There are many limitations to this study. Concerns include the small range of constructions being taught, the short-term nature of the experiment, the experimental environment and lack of ecological validity, the lack of long-term delayed testing, and the small range of outcome measures. The latter is a particular worry. As pointed out by one reviewer, attention/processing in this experiment is assessed through a considered comprehension temporal rating task, which likely taps predominantly explicit knowledge. Future research could well-incorporate a battery of measures ranging in their implicitness/explicitness. Meta-analyses of effects of instruction demonstrate that effectiveness varies as a result of explicitness of measure. Much also remains to be done particularly with regard to assessing the transfer of visual language experience to aural competence and vice-versa. We are currently comparing the effects of instruction under aural, visual, and bimodal conditions.

The findings in this study reinforce and extend prior studies in second language instruction. Specifically, in the absence of instruction, learners tend to ignore non-salient features in the input, such as verb morphology. FFI can increase the salience of inflections and other commonly ignored features by (i) explicitly identifying the forms and their functions, as in VG, (ii) by having the inflections made more salient by textual enhancement, as in VS, or (iii) by introducing the verb alone in a non-redundant context, as in VP. These are the type of techniques that help learners attend to verb morphology, and broadly, they do so to the same extent in the visual modality. However, our results demonstrate that the effectiveness of different types of FFI techniques in enhancing the salience and processing of these forms can vary as a function of modality of input presentation (i.e., aural or visual). Here, VG was effective across modalities, but VS was only advantageous in the visual modality. These findings should be considered in the design of optimal L2 instruction programs. Here, we only examined one specific type of construction, that of verb-tense morphology. We do not believe that the findings in this study will necessarily be true for all linguistic constructions. As the literature on FFI shows, different forms will require different levels of explicitness and explanation (Long, 2006, chap. 5; Spada and Tomita, 2010; Tolentino and Tokowicz, 2014).

Taken together, these findings demonstrate a range of effects of salience in L2 acquisition. Morphological forms are less well-attended than lexical forms. We believe that this reflects a combination of their relative psychophysical slightness in comparison to lexical cues, as well as effects of learned attention and blocking. There are effects of modality – attention to morphological cues is more effective from visual than from aural input. There are effects of instruction – form-focusing techniques such as grammar instruction, verb pretraining, or enhancing the salience of forms through typological or prosodic enhancement, can increase attention to these forms and increase processing. There are interactions between the effectiveness of FFI and modality such that typological salience enhancement from visual input is effective, while prosodic enhancement from aural input is not. Finally, brief EGI prior to language exposure is an effective means of raising the salience of otherwise ignored cues and turning input into intake. In learning a second language, some attention to form is necessary, and the forms that need to be attended are often the least salient in the input. Successful L2 acquisition rests on attention-focusing manipulations which raise the significance of otherwise non-salient cues.

## Author Contributions

The Abstract, Introduction and Discussion Sections were all contributed to equally by both authors. The methods section was written up by MC-V and edited by NE. The results were analyzed/written up by MC-V and edited by NE.

## Conflict of Interest Statement

The authors declare that the research was conducted in the absence of any commercial or financial relationships that could be construed as a potential conflict of interest.
